# Impacts of ammoniacal odour removal bioagent on air bacterial community

**DOI:** 10.1007/s44307-024-00016-w

**Published:** 2024-02-28

**Authors:** Hetian Zhang, Jin Hu, Xing Peng, Lei Zhou, Teng Zhang, Yanfang Zhang, Huaqun Yin, Delong Meng

**Affiliations:** 1 School of Minerals Processing and Bioengineering, Central South University, Changsha, 410083 China; 2 Key Laboratory of Biohydrometallurgy, Ministry of Education, Central South University, Changsha, 410083 China; 3 Hunan Renhe Environment Co., Ltd., Changsha, 410022 China; 4 Beijing Research Institute of Chemical Engineering and Metallurgy, Beijing, 101148 China

**Keywords:** Air microbial community, Bioagent, Air ammonia, Community assembly

## Abstract

While biotechnologies offer eco-friendly solutions for eliminating air contaminants, there is a scarcity of research examining the impacts of microbial purification of air pollutants on the structure and function of air microbial communities. In this study, we explored a *Lactobacillus paracasei* B1 (LAB) agent for removing ammoniacal odour. The impacts of LAB on air bacterial community were revealed. by analyzing the air samples before (BT) and after (AT) LAB bioagent treatment. Remarkably, the LAB bioagent significantly reduced the air ammonia concentration by 96.8%. This reduction was associated with a notable decline in bacterial diversity and a significant shift in community composition. The relative abundance of *Staphylococcus*, a common pathogen, plummeted from 1.91% to 0.03%. Moreover, other potential pathogens decreased by over 87%, signifying the bioagent's impactful role in diminishing health risks. The dominance of OTU-4 (*Lactobacillus*) highlighted its crucial role not only in competitive interactions but also potentially in shaping the metabolic pathways or community dynamics within the treated air microbial ecosystem. This shift towards deterministic assembly processes post-treatment, as highlighted by the normalized stochasticity ratio (NST), sheds light on the underlying mechanisms dictating the microbial community's response to bioagent interventions. The bioagent-purified air microbial community showed a strong preference for variable selection (88.9%), likely due to the acidity generated by the LAB. In conclusion, our findings emphasized the positive impact of LAB bioagent in enhancing air quality, which associated with the changes in microbial community.

## Introduction

Composting, a prevalent method converting organic waste into fertilizers (Sayara et al. 2020), confronts challenges in releasing odorous air, such as ammoniacal odour and harboring pathogenic bacteria like *Escherichia coli*, *Salmonella*, *Legionella*, and *Staphylococcus aureus* (Hong et al. 2012; Gao et al. 2023). Both ammoniacal odour and pathogenic bacteria in the air posed environmental and health risks (Qian et al. 2016), raising concerns about potential impacts related to odors and bioaerosols (Domingo and Nadal 2009; Robertson et al. 2019). Consequently, purifying compost air has become imperative. Various air purification technologies, including physical, chemical, and biological methods, have been explored in recent years to purifying the pollutants in composting air systems (Yan et al. 2014; Wysocka et al. 2019). Notably, air purifying biotechniques have gained lots traction for their adaptability, wide applicability, long-lasting effects, and simplicity, emerging as hot topics in current research (Kim et al. 2004; Kim and Park 2006). Microorganisms such as Yeast, *Lactobacillus*, and *Bacillus* utilized odorous substances as growth nutrients, producing enzymes like lipases and proteases and metabolites like metabolites, such as diacetyl and acetaldehyde (Chen et al. 2016; Ma et al. 2021). These microbial enzymes and metabolites possess aromatic properties that mask odors while inhibiting gas-producing and harmful microorganisms (Kim et al. 2021). Synthesized microbes refer to engineered or modified microbial strains often employed to enhance deodorization and bacteriostatic properties. For instance, co-culturing Yeast and *Lactobacillus* synergistically provides enhanced deodorization of ammonia and antibacterial effects through complementary nutritional mechanisms (Zhang et al. 2021b).

Bioremediation technologies have rapidly advanced, yet research on biotechnologies for air pollution control has been relatively limited compared to soil and water treatment. This limitation primarily stems from the lower concentration of gas molecules and microbial biomass in the air, compounded by challenges in sampling processes (Xie et al. 2021). However, understanding the relationship between air quality, microbial community, and human health is increasingly recognized as pivotal (Kan et al. 2012), and warrants further exploration. To comprehend the implications of air purifying technologies on microbial communities and elucidate potential health risks, comprehensive investigations are essential. These studies should delve into the mechanisms by how microbial agents impact air microbial communities, aiding the development of more effective bioagents.

In this study, the *Lactobacillus paracasei* B1 (LAB) was employed to purify air ammoniacal odour within a composting environment. We analyzed the air microbial community before and after LAB bioagent treatment by using the 16S rRNA gene high-throughput sequencing technology. The study aimed (i) to unravel how bioagents influenced the air microbial community during composting, (ii) to assess the role of LAB bioagents in mitigating air ammonia and pathogen risks, and (iii) to contribute valuable insights for the development of effective deodorizing bioagents.

## Materials and methods

### Preparation of bioagent

The bacterial strain used in this study was isolated from waste leachate and was identified as *Lactobacillus paracasei* B1. As reported in our previous study (Zhang et al. 2021b), the strain had good genetic and metabolic properties for removing air ammoniacal odour. The bacteria underwent fermentation to produce bioagents employed for air purification. The fermentation medium was consisted of 0.3 g/L glucose, 0.2 g/L yeast extract, 0.01 g/L KH_2_PO_4_ and 0.01 g/L MgSO_4_. The seed liquid was inoculated into 3 L of fermentation medium and fermented for 72 h in a 5 L tank reactor (BTF-A5L, Bio-Top Inc, Taichung, Taiwan) at 170 rpm and 35 ℃ for 24 h. Cell density was measured using a hemocytometer under a microscope (BX41, Olympus, Tokyo, Japan). After 72 h of cultivation, when *L. paracasei* B1 reached a stable phase, the resulting fermentation was diluted and then utilized as a bioagent for air ammonia purification. The pH of the fermentation was determined using a pH meter (Remagnet PHS-3C, Shanghai China), and the pH was 3.22. For the air purification treatment, the bacterial concentration was diluted to 1 × 10^8^ CFU/ml before use by mixing the fermentation broth with distilled water at a 1:50 ratio.

### Air sample collection

For sludge composting, raw materials were well thoroughly mixed and formed into a conical heap weighing 155 kg, with a bottom diameter of 1.5 m and a height of approximately 1 m. The aerator was positioned around 0.3 m from the pile's bottom, providing aeration at a rate of 4 L/min (Zhang et al. 2021a). The bioagent was sprayed in a 3 m radius from the heap's center using an atomizing sprayer. The spray rate was set at 50 ml/min, totaling 1L sprayed over a 20-min duration. This spraying procedure and volume were consistent with similar deodorants available in the market.

**Fig. 1:**
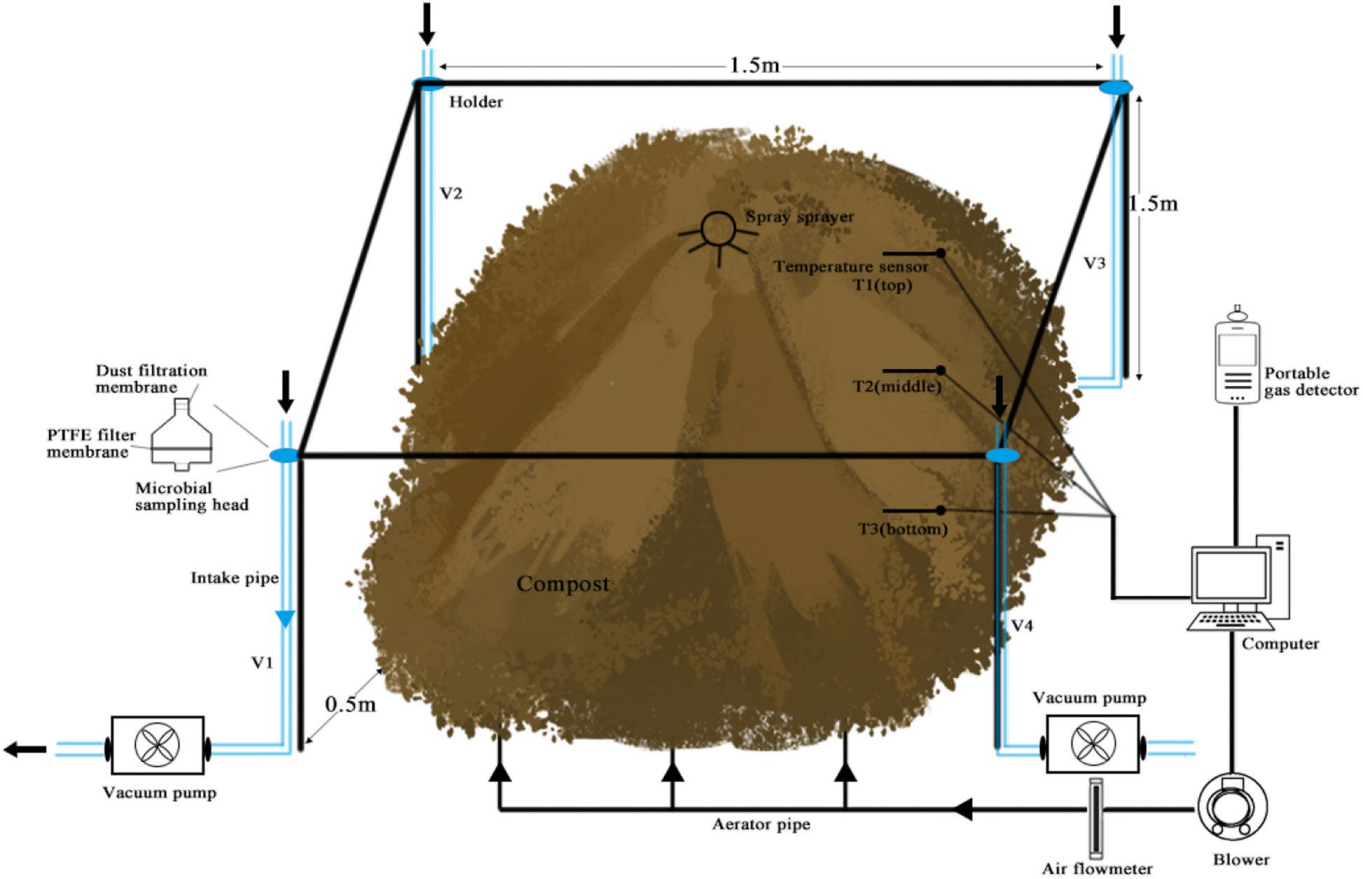
A scheme figure showing the experimental design, and air sample collection

Air samples were collected before (BT) and after (AT) bioagent treatment, positioned 1.5 m above the sludge compost (Fig. 1). Air microorganisms were collected using polytetrafluoroethylene (PTFE) filters (47 mm in diameter and 0.45 μm pore size, sourced from China) with a vacuum pump. Four strategically placed vacuum pumps—front, back, left, and right of the pile—collected samples at a flow rate of 21 L min^−1^ for 15 min each (Bekking et al. 2019). The filter membranes containing microorganisms were used for DNA extraction, and this process was repeated for 10 times to obtain enough replications. To measure ammonia concentration above the sludge compost, a portable gas analyzer (Eranntex MS400, China) was employed over a 3-h duration. Immediately after applying the biological agent, the vacuum pump was activated for sampling. The sampling method and repetition mirrored the pre-treatment sampling, resulting in a total of 10 AT samples.

### DNA extraction, PCR amplification, sequencing, and data processing

The polytetrafluoroethylene (PTFE) filter membranes were first cryogenically fractured in liquid nitrogen. Total DNA was extracted from each sample using FastDNA SPIN Kit for Soil (Mpbio, CA, USA) following the manufacturer’s instructions. The quantity and quality of DNA were examined using a Nanodrop 1000 spectrophotometer (Thermo Fisher Scientific, CA, USA). The primers of 341F (5’-CCTACGGGNGGCWGCAG-3’) and 805R (5’-GACTACHVGGGTATCTAATCC-3’) were used for the amplification of the V3-V4 region of the microbial 16S rRNA gene sequence (Hugerth et al. 2014). The amplified 16S rRNA gene was sequenced and analyzed using an Illumina MiSeq at LC Sciences (Hangzhou, China).

Sequence processing was conducted using the Galaxy pipeline (http://zhoulab5.rccc.ou.edu:8080/root). Initially, all sequences were sorted into samples based on their unique barcodes. Only sequences containing primers were retained, with subsequent removal of the primer sequences from the amplicons. The software Flash (v1.0.0) was employed to merge the forward and reverse sequences. To ensure data quality, reads with a QC score < 20 and a length < 140 bp were trimmed using Btrim (v1.0.0) (Yong 2011). Following this, the Trim N tool (v1.0.0) was used to eliminate sequences before the 'N' base and retain the sequences after 'N'. Additionally, reads with a total length less than 200 bp were removed, and the remaining reads were further trimmed to a length between 260–270 bp. Operational taxonomic units (OTUs) were clustered with a 97% similarity threshold using UPARSE (v7.0.1001) (Edgar 2010). Subsequently, the taxonomic classification of OTU sequences was performed through the RDP Classifier at a confidence level of 50% (Bacci et al. 2015). The resulting clean read data were deposited in the NCBI Sequence Read Archive (SRA) database under the BioProject accession number SUB11333698.

To justify the OTU assignment of LAB bioagent, we also include a bioagent DNA sample for 16S rRNA high through-put sequencing. The bioagent sample was consisted of OTU-4. In addition, the OTU-4 can be classified to genus *Lactobacillus*. Therefore, the OTU-4 can represent the bacterial strain, namely *Lactobacillus paracasei* B1, in bioagent.

Community analyses including the α-diversity and β-diversity calculations were performed on R statistical platform by using the *vegan* package (version 2.4–1).

### PICRUSt2 functional prediction

The microbial metabolic function was predicted using PICRUSTs (http://picrust.github.com/picrust/, phylogenetic investigation of communities by reconstruction of unobserved states) by referencing the KEGG (Kyoto Encyclopedia of Genes and Genome) Orthology database (Douglas et al. 2020). STAMP (v.2.1.3) was used to analyze and show the predicted relative abundances of gene functions (Langille et al. 2013).

### Molecular ecological network construction

The molecular ecological networks were constructed for both BT and AT samples. To clarify the ecological role of bioagent strain, we constructed the subnetwork of OTU-4. The molecular ecological network was constructed using CoNet (Co-occurrence Network, http://apps.cytoscape.org/apps/conet/) method. The construction process of the co-occurrence network is as follows: (i) preprocessing (filtering, normalization) to account for sequencing depth differences, set ‘*row minocc’* to half the total number of samples, and standardization select column; (ii) select from several different correlations, similarities or dissimilarities to score the association strength between the objects. set the edge number (140) off before treating and the quantile (0.2) after treating as the threshold setting (initial network); (iii) computation of permutation and bootstrap distributions (final network with *p*-values), select ''*edgeScores*'' as a routine and ''*shuffle_rows*'' as a resampling strategy, and enable *''Renormalize and Save randomizations to file*''. ''*Bootstrap*'' was selected as the recovery method, and ''*brown*'' was used as the *p*-value merge strategy (Faust and Raes 2016). Cytoscape software was used to visualize the network (Cline et al. 2007).

### Stochasticity and determinism in community assembly analysis

A null-model mathematical framework was used to calculate the relative contribution of the deterministic and stochastic processes to community assembly. Normalized Stochasticity Ratio (NST) based on null-model analysis in this frame was used to measure the position of observations under purely deterministic and purely stochastic conditions. The software R package calculates the NST index, and the corresponding R package is obtained from https://cran.r-project.org/package=NST. NST < 50% indicates deterministic processes; the deterministic process has more contribution to community assembly. On the contrary, NST > 50% indicates stochastic processes being more significant (Ning et al. 2019).

To calculate the contribution of ecological processes, the phylogenetic tree of all OTUs was first constructed using Usearch. For further phylogenetic analysis, we calculated βMNTD and βNTI based on a zero-model for system development and classification to test which community assembly process can best explain microbial community assembly. The βMNTD represents the difference between communities, and the negative value of the difference between βMNTD and the average of zero distribution is called βNTI. The replacement of phylogenetic community composition was characterized by βMNTD, and RC bray characterized the replacement of OTU composition. βNTI and Bray–Curtis-based Raup-Crick (RC) were used to quantify the contribution of major ecological processes to bacterial assembly communities (Stegen et al. 2012). When |βNTI|> 2, the community assembly is driven by a deterministic process, such as homogeneous selection (βNTI < -2) and variable selection((βNTI > 2). When (|βNTI|< 2), community has been assembled by a stochastic process, including homogenization diffusion (RC < -0.95), dispersal limitation (RC >  + 0.95), and drift (and others) (|RC|> + 0.95) (Stegen et al. 2012).

## Results

### Ammoniacal odour removal efficiency

**Fig. 2:**
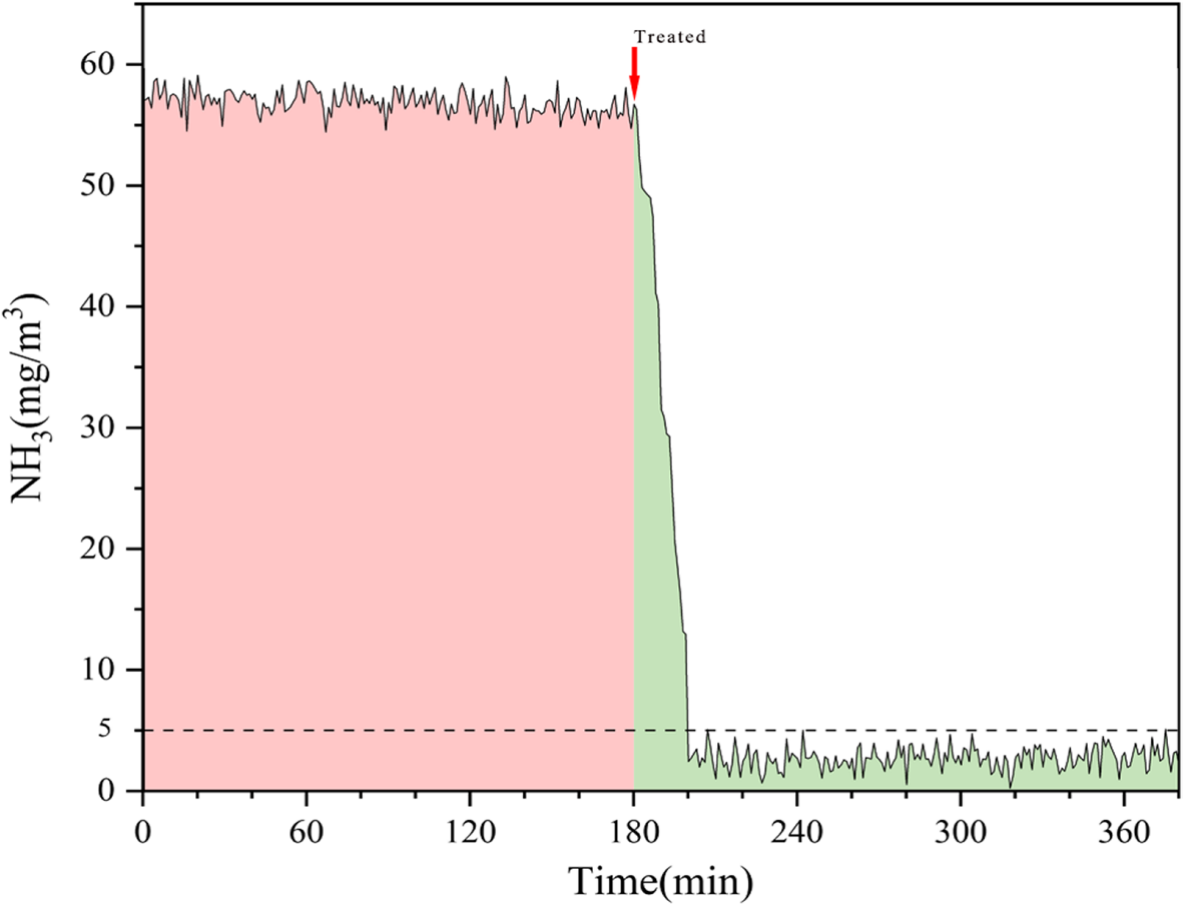
Change in air ammonia concentration before and after the treatment of bioagent. The red and green areas represent the ammonia concentration before and after treatment. The reference line is the ammonia concentration limit in the secondary standard for malodorous pollutants

Microbial ammonification in compost can result in a considerable amount of ammonia being released, which is a typical odor gas. During the high temperature stage of compost, the ammonia concentration in the composting air was about 57.1 mg/m^3^. The change in air ammonia concentration before and after treatment with LAB bioagent is shown in Fig. 2. The bioagent reduced atmospheric ammonia from 57.1 mg/m^3^ to 1.81 mg/m^3^ within 20 min. The ammonia removal efficiency of the bioagent was 96.8%, and the purifying effect could last for more than 3 h.

### Atmospheric bacterial community diversity and structure

**Fig. 3:**
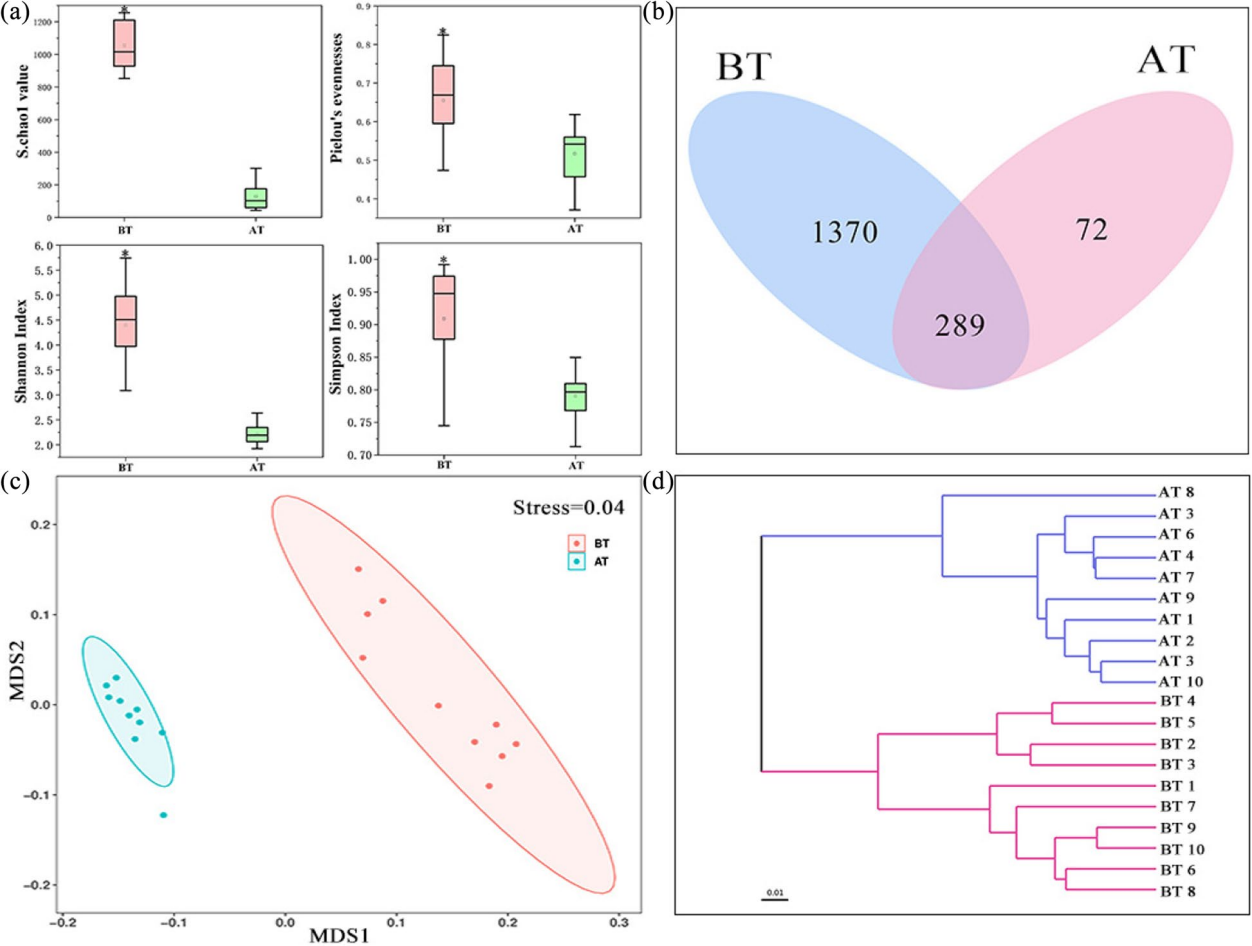
Overview of changes of bacterial community diversity in air before and after bioagent treatment. **a** α-diversity indices, including S.chao1, Pielou’s evenness, Shannon index and Simpson index, **b** Venn diagram, **c** The nonmetric multidimensional scaling (NMDS) analysis, stress = 0.04; **d** UPGMA cluster tree

We used 16S rRNA high-throughput sequencing approach to investigate the diversity and structure of bacterial communities before and after treatment. After pruning and removing low-quality reads, a total of 740, 322 high-quality 16S rRNA sequences were identified in two treatment groups (20 samples), resulting in 1792 OTUs. The Shannon index, Simpson index, Pielou’s evenness, and Chao 1 value all differed between the two groups, bacterial diversity of BT were higher than AT (Fig. 3a).

In the NMDS plot, the AT samples were significantly separated from the BT samples, demonstrating obvious differences in microbial community composition between two groups (Fig. 3c). According to UPGMA cluster analysis, the community composition of the two groups differed significantly. The Venn diagram showed the BT samples (1659) contained more OTUs than the AT samples (461), and the BT samples (87.3%) had a high proportion of unique OTUs (Fig. 3b).

**Fig. 4:**
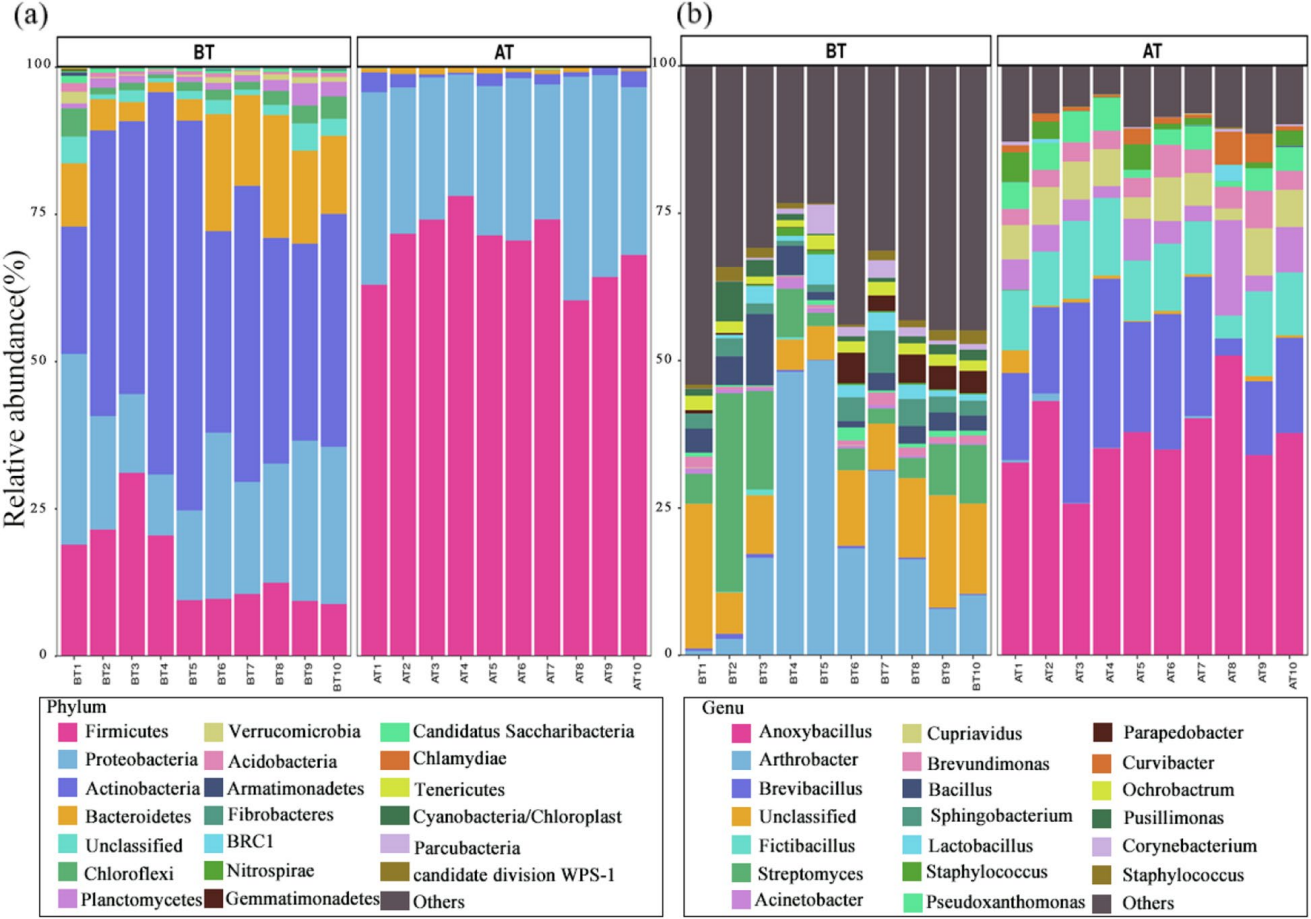
Changes in relative abundance of atmospheric microbial community before and after bioagent treatment. (**a**) at the phylum level; (**b**) at the genus level

Before and after treatment, there were significant variations in the proportions of each phylum. The dominant phylum in the BT group were Actinobacteria (44.34%), Proteobacteria (21.19%), Firmicutes (15.26%), and Bacteroidetes (10.98%). Firmicutes (69.62%) had a substantial increase in relative abundance after treatment with LAB bioagents, but Actinomycetes (1.63%) and Bacteroidetes (0.74%) had a significant decrease (Fig. 4.a). Changes in the microbial community composition at the genus level were noticeable and similar to those seen at the phylum level. The relative abundance of *Arthrobacter* (from 20.21% to 0.22%) and *Streptomyces* (from 35.9% to 9.41%) in Actinobacteria declined dramatically, while the relative abundance of *Anoxybacillus* in Firmicutes increased from 0% to 37.24%. The relative abundance of typical human pathogens, *Staphylococcus aureus*, decreased from 1.91% to 0.03%, while the abundance of other potential pathogens, *Corynebacterium*, *Bacillus*, *Ochrobactrum*, and *Spingobacterium*, also decreased by more than 87%, lowering the health risk associated with air pollution (Fig. 4b).

### Microbial community function

**Fig. 5:**
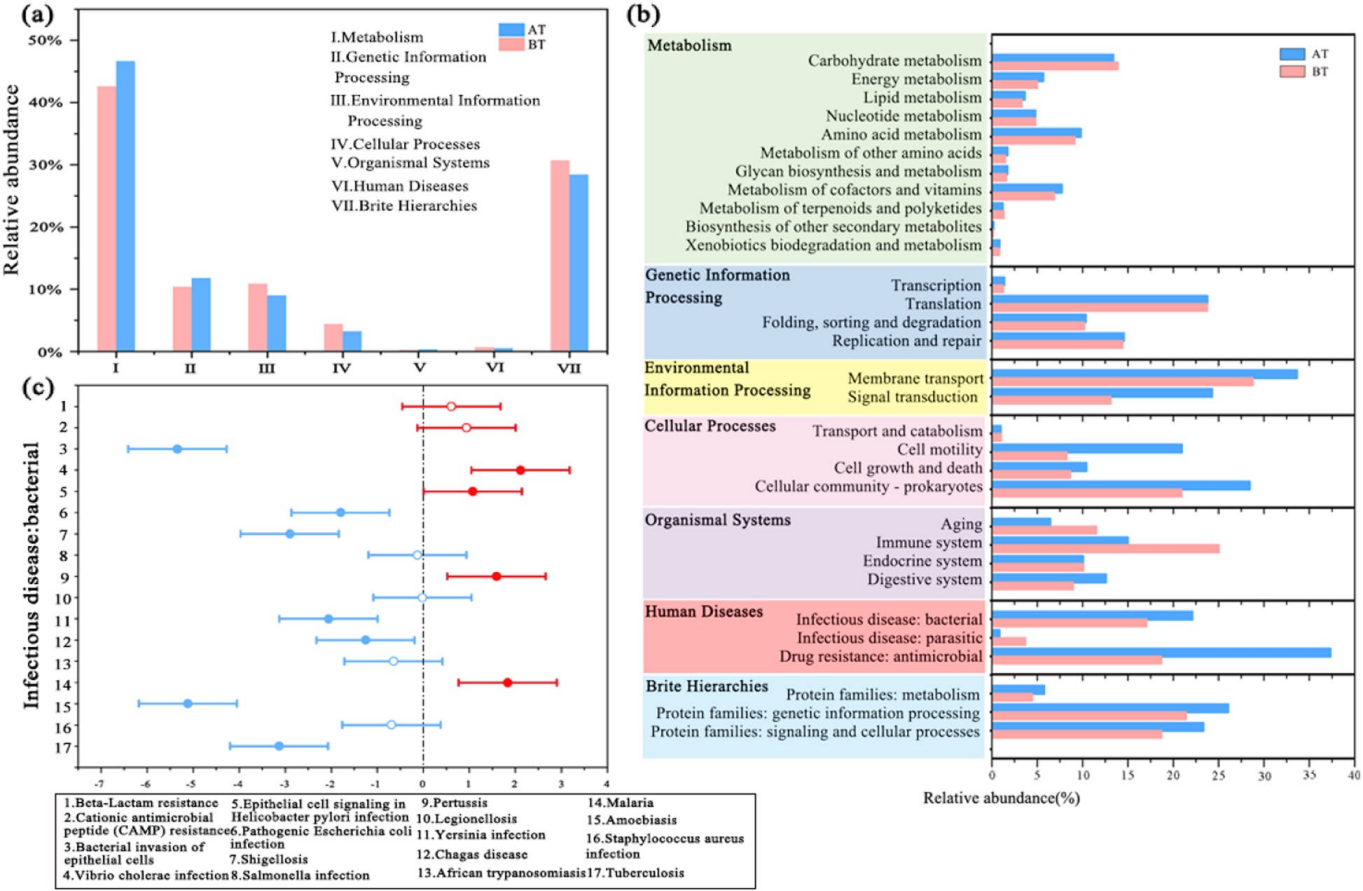
KEGG enrichment analysis based on PICRUSt analysis (**a**) Biochemical metabolic pathways; (**b**) level 2; (**c**) Response ratio of KEGG functions associated with bacterial infections to bioagent treatment

The changes observed in the microbial community structure corresponded with functional succession. In this study, we utilized PICRUST2 to predict the microbial community function. The predominant function within the air microbial community was metabolism, exhibiting an increase from 42.62% to 46.66%. This was followed by genetic information processing (from 10.41% to 11.81%), environmental information processing (from 9.03% to 10.90%), cellular processes (from 3.23% to 4.41%), and human diseases (from 0.51% to 0.69%) (Fig. 5a). In terms of metabolic pathways, carbohydrate metabolism was more pronounced in the pre-treatment (BT) phase, while energy metabolism, lipid metabolism, amino acid metabolism, metabolism of cofactors, vitamin metabolism, and biosynthesis of other secondary metabolites were lower, than in AT. In the environmental information processing category, membrane transport was higher in BT, whereas signal transduction was increased in the post-treatment (AT) phase (Fig. 5b). Additionally, cell motility in cellular processes displayed a decrease following bioagent treatment. Moreover, a detailed investigation focused on alterations in genes associated with bacterial infection at the third level, employing response ratio analysis. The results indicated a decrease in the abundance of predicted genes linked to infectious diseases such as those affecting the respiratory tract, intestinal tract, and skin in AT samples. Notably, the LAB bioagent exhibited significant inhibition of bacterial invasion of epithelial cells, Salmonella infection, Yersinia infection, American trypanosomiasis, and tuberculosis (Fig. 5c).

### Air molecular ecological network

**Fig. 6:**
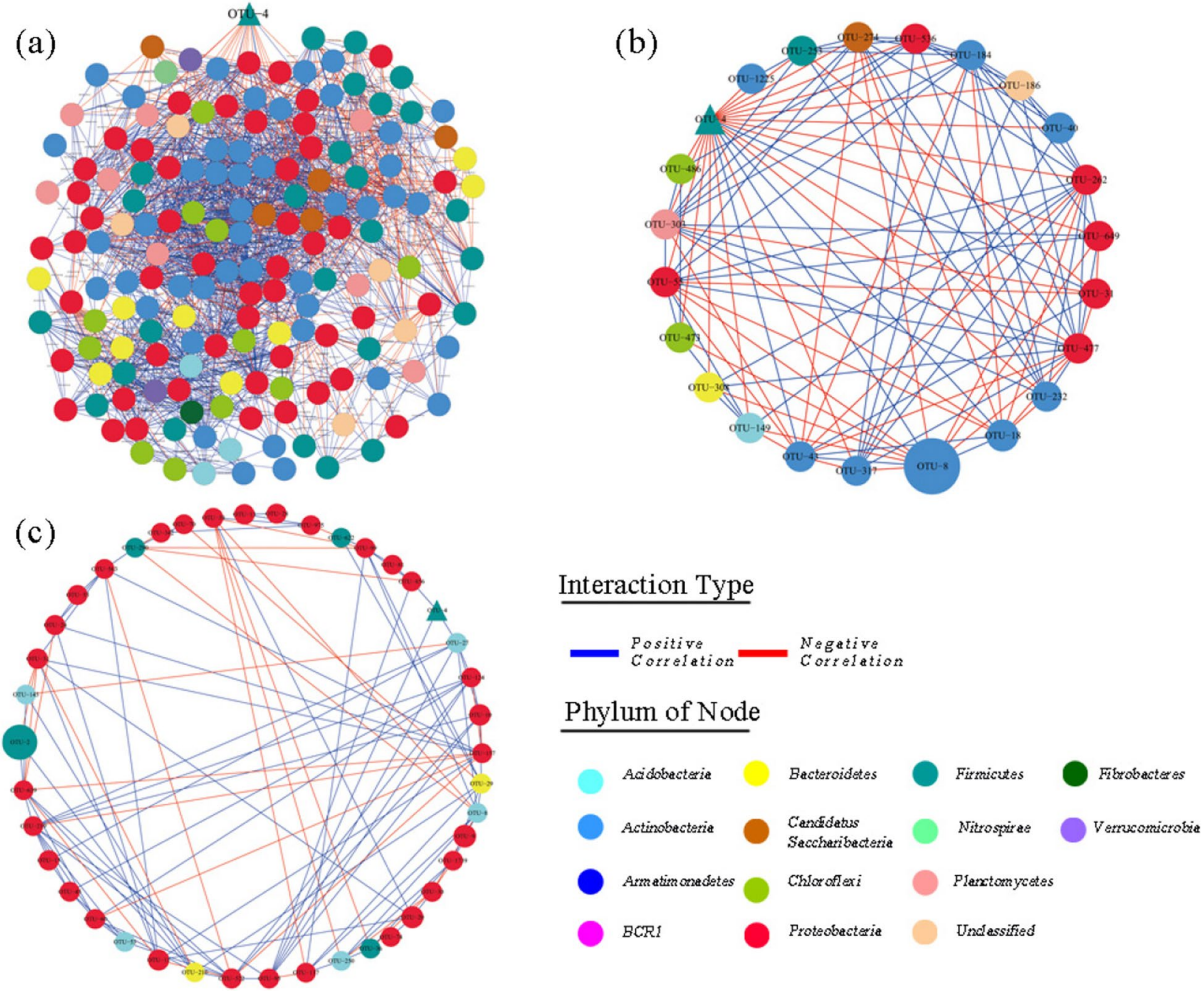
Molecular ecological network of air bacterial community. (**a**) before bioagent treatment; (**b**) subnetworks of OTU-4 (*Lactobacillus*) before treatment; (**c**) after bioagent treatment. Each edge represents an important co-occurrence relationship. The edge is colored by correlation: the positive correlation is blue, and the negative correlation is red. The node size corresponds to the abundance of each OTU, and the color corresponds to the category taxonomy. The OTU-4 represents the bacterial strain in agent

The molecular ecological network results (Fig. 6) highlighted distinct characteristics between the microbial molecular networks before and after ammonia removal bioagent treatment. The network observed before treatment displayed complexity and compactness, encompassing 439 nodes and 8342 connecting lines. It was predominantly composed of positive interactions, accounting for 80.04% of the total interactions. In contrast, the post-treatment (AT) network exhibited a comparatively basic structure, featuring 42 nodes and 107 connecting lines, still leaning towards positive interactions (71.96%). Notably, a pronounced competitive interaction surfaced between the *Lactobacillus* strain (OTU-4) and other microbes. Within the BT network, *Lactobacillus* (OTU-4) demonstrated a substantial negative correlation with its network neighbors, reaching 86.36%. This suggests a robust competitive or antagonistic relationship between *Lactobacillus* (OTU-4) and other species. These findings suggest a dual effect of *Lactobacillus* within the bacterial community environment. On one hand, its presence led to alterations beneficial to the environment, such as reducing ammonia concentration in the air and lowering the relative abundance of pathogenic microorganisms. However, these negative interactions, primarily competition, contributed to a restructuring of the network from a complex and cohesive structure to a simpler and looser arrangement.

### The assembly process of air microbial community

**Fig. 7:**
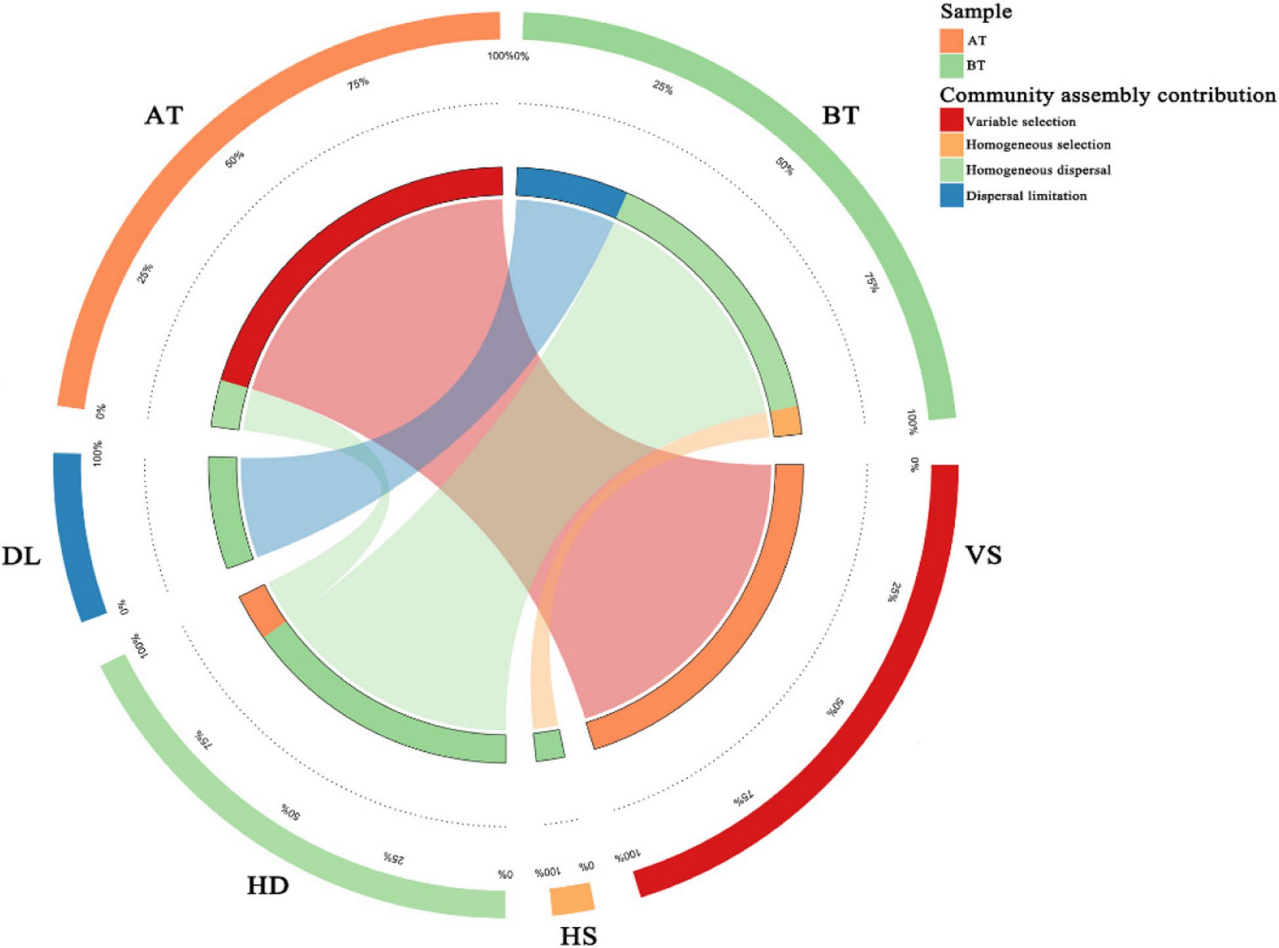
Relative contribution of deterministic and stochastic assembly processes on air communities before and after treatment

To further investigate the role of ecological processes in shaping the air microbial community, we employed two types of null model-based analyses: a) Standizing the null model-based normalized stochastic ratios (NST) using Bray–Curtis metrics. NST uses 50% as the boundary point. When NST is greater than 50%, it indicates that stochasticity contributes more to community assembly, whereas determinacy plays a dominant role. The NST of BT and AT was 38.14% and 4.40%, respectively. The estimated value of NST decreased as the complexity of the community decreased, and the assembly of air bacterial communities before and after treatment shifted from a deterministic process to a more deterministic process. b) Quantifying community assembly processes using a phylogenetic β-diversity metric (β-NTI) and taxonomic β-diversity metrics (Bray–Curtis-based Raup-Crick, RC_bray_). Based on β-NTI and RC_bray_, the community assembly was classified as homogeneous selection, variable selection, dispersion limitation, homogenizing dispersal, and undominated processes. After treatment, |β-NTI| of the samples changed from < 2 to > 2, indicating that the community assembly evolved from a stochastic process to a deterministic process, which was relatively consistent with NST. The community assembly of BT was dominated by homogeneous diffusion (66.7%), followed by diffusion limitation accounting for 26.7%. The AT of community assembly was dominated by the selection process, with variable selection accounting for 88.9% (Fig. 7).

## Discussion

### Bioagents have shown effectiveness in efficiently and enduringly removing ammonia from composting air

*L. Paracasei* B1 is known to produce lactic acid, butyric acid, and other metabolites during fermentation, resulting in a final fermentation product with a pH of 3.22. This acidic environment facilitated the rapid decrease of ammonia concentration in the air by triggering an acid–base neutralization reaction between H^+^ and NH_3_, forming ammonium dissolved in water. Additionally, the residual agent formed a protective layer over the compost surface, creating a physical canopy that effectively captures ammonia emissions, reducing nitrogen loss over an extended period. One concern raised was the potential disturbance to the compost microbial system caused by the deodorant spraying. However, *L. Paracasei* B1 as an additive not only regulated ammonia emissions positively but also contributed to enhancing the quality of the reactor. At higher temperatures and increased pH levels, NH_4_^+^/NH_3_ assimilation was accelerated. While certain additives like calcium superphosphate, sulfuric acid, and phosphoric acid effectively reduced ammonia emissions in the composting process, they significantly inhibited microbial activity (Hui and Huang 2009). The fermentation broth of lactic acid bacteria acts as an appropriate pH-regulating buffer, creating a conducive weak acid environment in the compost that reduces ammonia levels while continuing to be degraded and utilized by microorganisms. Extensive studies have demonstrated the significant impact of lactic acid bacteria fermentation products on deamination within composting systems, promoting compost (Nie et al. 2020; Zhu et al. 2020).

### The bioagent used for odor removal significantly decreased the diversity of the air microbial community by suppressing the growth of other microbes

The handling of solid waste is a crucial contributor to airborne microorganisms in composting (Wery 2014; Fang et al. 2023). Operations like waste crushing, transportation, and aeration during stacking in the fermentation process disperse numerous biological components into the surrounding air (Bonifait et al. 2017). Our study showed that the air microbial communities before bioagent treatment were dominated by Actinomycetes and Firmicutes (Fig. 4), which was consistent with previous research (Goff et al. 2010). Moreover, the application of LAB bioagents significantly altered the air microbial community, evident in reduced diversity, altered composition, and structural changes. These alterations resulted from the suppression of certain microbial groups by the agents, illustrated by the Venn diagram indicating a considerable portion of OTUs (1370 out of 1659) absent in AT samples compared to BT samples. The bioagents suppressed microbes (OTUs) in BT samples through two mechanisms: the potent selection effects of highly acidic (pH = 3.22) LAB agents and the competitive effects of the *Lactobacillus* strain. The bioagents lowered air pH, neutralizing airborne ammonia (Zhang et al. 2021b). The lowered pH effectively filtered most air microorganisms, directly impacting the air microbial community's diversity and composition. This is further supported by the fact that most compost microorganisms thrive in mildly alkaline environments but struggle or perish in acidic conditions (Chen et al. 2012). Specifically, the species and abundance of Actinomycetes in compost were shown to be negatively impacted by soil acidity. Additionally, there was a significant decline in the abundance of Actinomycetes such as *Arthrobacter* and *Streptomyces* (Fig. 4).

Network analysis revealed a strong negative correlation between *Lactobacillus* (OTU-4) and the majority of nodes, indicating a competitive relationship between Lactobacillus and air microorganisms. This competition may be attributed to three key aspects: (1) Resource scarcity: The spraying increased Lactobacillus density in the air, competing with indigenous bacterial communities for limited nutrients (Siedler et al. 2020). (2) Metabolite antagonism. *Lactobacillus* generated organic acids such as lactic acid and acetic acid, which can not only create an acidic environment but also alter the permeability of bacterial cell membranes, disrupting normal physiological metabolism. The decrease in membrane transport after bioagent treatment may be related to the inhibition of organic acids (Fig. 5b). Therefore, *Lactobacillus* was widely used in food preservation (Gharsallaoui et al. 2016). (3) Antimicrobial effects. *Lactobacillus* generated a variety of bacteriocins and antimicrobial peptides to inhibit microorganisms, which have a wide antimicrobial spectrum and a good inhibitory effect on *Salmonella* and *E. coli* (Reis et al. 2012; Wang et al. 2015). The enhanced metabolic capacity of lipids, amino acids, cofactors, and secondary metabolites may be a manifestation of promoting the synthesis of resistance factors to improve their resistance to adverse environment conditions (Fig. 5b).

### The bioagents effectively lowered pathogen levels, suggesting a potential reduction in air health risks

Our study found that LAB bioagent not only altered the microbial community structure but also lowered the relative abundance of pathogenic bacteria. The abundance of typical pathogenic bacteria, *Staphylococcus aureus*, in AT samples decreased significantly after bioagent treatment (Fig. 4). *S. aureus* was the most common pathogen, and reports of poisoning caused by enterotoxins emerge endlessly it produces abound (Kadariya et al. 2014). In addition, *S. aureus* in the human bloodstream or tissue can cause a range of potentially serious infections (Tuchscherr et al. 2011). *S. aureus* had high morbidity and mortality, especially methicillin-resistant *Staphylococcus* infection caused by clinical health threats to public health. *Lactobacillus* can produce acetic acid and lactic acid to inhibit the growth activity of *S. aureus*, and block the spread by affecting the adhesion of cell biofilm by bacteriocin and catalase to reduce adhesion. It has become a feasible choice to prevent *S. aureus*, which is consistent with the decrease in *S. aureus* abundance caused by LAB bacteria. *Lactobacillus* has been widely used to inhibit *S. aureus,* and it was generally used to inhibit the formation of biofilm and induce ultrastructural changes through bacteriocin and other metabolites (Rybalchenko et al. 2010; Lavryk et al. 2017). In addition, the potential opportunistic pathogenic bacteria *Sphingobacterium*, *Corynebacterium*, *Bacillus*, and *Streptomyces* in AT samples were lower than those in BT samples. These genera had low virulence, while some species are quite virulent. The LAB agent had a considerable inhibitory effect on these pathogens as well. We believed this was related to the abundant natural antimicrobial metabolites in LAB bioagent. Functional gene prediction showed a significant reduction in genes associated with respiratory infectious diseases, intestinal infectious diseases, and parasitic infectious diseases, which were associated with *Salmonella* infection, Legionnaires' disease, Chagas disease, and tuberculosis. This demonstrated that bioagent could eliminate pathogens in the composting air, lowering the danger of exposure to pathogens for compost workers. The widespread use of *Lactobacillus* in the food and pharmaceutical industries Studies have also shown *Lactobacillus* was an important probiotic related to human beings (Ritchie and Romanuk 2012). It was showed that *Lactobacillus* actively antagonizes pathogenic bacteria in the human respiratory tract and digestive tract (Mu et al. 2018). Our study also indicated that *Lactobacillus* could be used to prevent and inhibit the spread of pathogenic bacteria in the air, and therefore reduced air pathogenic risks. This indicated that LAB not only controlled odor from the source of odor but also had feasibility in the field of disinfection.

### The LAB bioagent reinforced deterministic processes in the assembly of the air community

The application of bioagents resulted in a shift towards a more deterministic assembly model of the air microbial community, prominently influenced by specific environmental pressures introduced by the LAB bioagent (Fig. 7) (Starnawski et al. 2017). This shift towards deterministic processes, particularly variable selection, suggested a community structure driven by distinct environmental factors, possibly stemming from the selection, competition, and antagonistic effects induced by *L. paracasei* B1.

Variable selection, shaped by biological or abiotic environmental conditions, can significantly alter microbial community structures (Zhang et al. 2019). In our study, the influence of biological agents acting as environmental pressures instigated substantial changes in the air microbial communities. This phenomenon is akin to observations in aquatic environments, where nutrient levels dictate the assembly of planktonic bacteria. Here, specific nutrient conditions prompt a strong community response, selecting species that can thrive in the given environment after environmental filtration (e.g., suspended solids concentration and pH). Similarly, following LAB agent treatment, only adaptable microorganisms could successfully colonize and perform ecological functions within the ecosystem (Hanashiro et al. 2022). The strong influence of variable selection can lead to biological homogenization, wherein community compositions become more alike (Monchamp et al. 2018), This process might have also impeded colonization for many species. Maintaining this environmental pressure necessitated regular application of LAB bioagents to sustain these selective conditions.

## Conclusions

In summary, our study comprehensively explored the varied impacts of the *L. paracasei* B1 deodorant on the air microbial community. We explored a bioagent consisted of lactic acid bacteria (LAB), which could reduce air ammonia by 96.8% and sustained the effect for over 3 h. The results also demonstrated that the LAB bioagent induced changes in the microbial community structure, leading to reduced diversity, alterations in taxonomic composition, and shifts in microbial interactions. This bioagent effectively suppressed the spread of pathogens in the air, mitigating health risks associated with air pollution. Introducing the LAB bioagent exerted significant environmental pressure, influencing the assembly of the microbial community. Collectively, our findings shed light on the multifaceted effects of LAB bioagents on air communities, thereby advancing their application and development for purifying composting air.

## Data Availability

The clean read data of 16S rRNA sequencing were deposited in the NCBI Sequence Read Archive (SRA) database under the BioProject accession number SUB11333698.

## References

[CR1] Bacci G, Bani A, Bazzicalupo M, Ceccherini MT, Galardini M, Nannipieri P, et al. Evaluation of the Performances of Ribosomal Database Project (RDP) classifier for taxonomic assignment of 16S rRNA metabarcoding sequences generated from illumina-solexa NGS. J Gen. 2015;3:36–9. 10.7150/jgen.9204 PMC4316179 25653722

[CR2] Bekking C, Yip L, Groulx N, Doggett N, Finn M, Mubareka S. Evaluation of bioaerosol samplers for the detection and quantification of influenza virus from artificial aerosols and influenza virus-infected ferrets. Influenza Other Respir Viruses. 2019;13(6):564–73. 31541519 10.1111/irv.12678 PMC6800310

[CR3] Bonifait L, Marchand G, Veillette M, et al. Workers’ exposure to bioaerosols from three different types of composting facilities. J Occup Environ Hygiene. 2017;14(10):815–22. 10.1080/15459624.2017.1335054 28636488

[CR4] Chen W, Li Z-W, Shen X. Influence of soil acidification on soil microorganisms in pear orchards. Commun Soil Sci Plant Anal. 2012;43(13):1833–46.

[CR5] Chen D, Chen X, Chen H, Cai B, Wan P, Zhu X, et al. Identification of odor volatile compounds and deodorization of Paphia undulata enzymatic hydrolysate. J Ocean Univ China. 2016;15(6):1101–10.

[CR6] Cline MS, Smoot M, Cerami E, Kuchinsky A, Landys N, Workman C, et al. Integration of biological networks and gene expression data using cytoscape. Nat Protoc. 2007;2(10):2366–82. 17947979 10.1038/nprot.2007.324 PMC3685583

[CR7] Domingo JL, Nadal M. Domestic waste composting facilities: a review of human health risks. Environ Int. 2009;35(2):382–9. 18701167 10.1016/j.envint.2008.07.004

[CR8] Douglas GM, Maffei VJ, Zaneveld JR, Yurgel SN, Brown JR, Taylor CM, et al. PICRUSt2 for prediction of metagenome functions. Nat Biotechnol. 2020;38(6):685–8. 32483366 10.1038/s41587-020-0548-6 PMC7365738

[CR9] Edgar RC. Search and clustering orders of magnitude faster than BLAST. Bioinformatics. 2010;26(19):2460. 20709691 10.1093/bioinformatics/btq461

[CR10] Fang R, Chen T, Han ZB, Ji WH, Bai YD, Zheng ZP, et al. From air to airway: dynamics and risk of inhalable bacteria in municipal solid waste treatment systems. J Hazard Mater. 2023;460:132407. 37651934 10.1016/j.jhazmat.2023.132407

[CR11] Faust K, Raes J. CoNet app: inference of biological association networks using Cytoscape. F1000 Res. 2016;5:1519. 10.12688/f1000research.9050.1 PMC5089131 27853510

[CR12] Gao F-Z, He L-Y, He L-X, Bai H, Zhang M, Chen Z-Y, et al. Swine farming shifted the gut antibiotic resistome of local people. J Hazard Mater. 2023;465:133082–133082. 38016315 10.1016/j.jhazmat.2023.133082

[CR13] Gharsallaoui A, Oulahal N, Joly C, Degraeve P. Nisin as a food preservative: part 1: physicochemical properties, antimicrobial activity, and main uses. Crit Rev Food Sci Nutr. 2016;56(8):1262–74. 25675115 10.1080/10408398.2013.763765

[CR14] Goff OL, Bru-Adan V, Bacheley H, Godon J, Wéry N. The microbial signature of aerosols produced during the thermophilic phase of composting. J Appl Microbiol. 2010;108(1):325–40. 19602015 10.1111/j.1365-2672.2009.04427.x

[CR15] Hanashiro FTT, De Meester L, Vanhamel M, Mukherjee S, Gianuca AT, Verbeek L, et al. Bacterioplankton assembly along a eutrophication gradient is mainly structured by environmental filtering, Including Indirect Effects of Phytoplankton. Composition. 2022;85(2):400–10. 10.1007/s00248-022-01994-x 35306576

[CR16] Hong PY, Li X, Yang X, Shinkai T, Zhang Y, Wang X, et al. Monitoring airborne biotic contaminants in the indoor environment of pig and poultry confinement buildings. Environ Microbiol. 2012;14(6):1420–31. 22414212 10.1111/j.1462-2920.2012.02726.x

[CR17] Hugerth LW, Wefer HA, Lundin S, Jakobsson HE, Lindberg M, Rodin S, et al. DegePrime, a program for degenerate primer design for broad-taxonomic-range pcr in microbial ecology studies. Appl Environ Microbiol. 2014;80(16):5116–23. 24928874 10.1128/AEM.01403-14 PMC4135748

[CR18] Hui Y, Huang GH. Effects of sodium acetate as a pH control amendment on the composting of food waste. Biores Technol. 2009;100(6):2005–11. 10.1016/j.biortech.2008.10.007 19042126

[CR19] Kadariya J, Smith TC, Thapaliya D. *Staphylococcus aureus* and staphylococcal food-borne disease: an ongoing challenge in public health. Biomed Res Int. 2014;2014:827965. 24804250 10.1155/2014/827965 PMC3988705

[CR20] Kan HD, Chen RJ, Tong SL. Ambient air pollution, climate change, and population health in China. Environ Int. 2012;42:10–9. 21440303 10.1016/j.envint.2011.03.003

[CR21] Kim JD, Park KM. Effectiveness of *Lactobacillus plantarum* strain KJ-10311 to remove characteristic malodorous gases in piggery slurry. Asian Australas J Anim Sci. 2006;19(1):144–52.

[CR22] Kim TI, Ham JS, Yang CB, Kim MK. Deodorization of pig feces by fungal application. Asian Australas J Anim Sci. 2004;17(9):1286–90.

[CR23] Kim MJ, Tagele SB, Jo H, Kim MC, Jung Y, Park YJ, et al. Effect of a bioconverted product of Lotus corniculatus seed on the axillary microbiome and body odor. Sci Rep. 2021;11(1):10138. 33980951 10.1038/s41598-021-89606-5 PMC8115508

[CR24] Langille M, Zaneveld J, Caporaso JG, Mcdonald D, Knights D, Reyes JA, et al. Predictive functional profiling of microbial communities using 16S rRNA marker gene sequences. Nat Biotechnol. 2013;31(9):814–21. 23975157 10.1038/nbt.2676 PMC3819121

[CR25] Lavryk G, Korniychuk O, Tymkiv M. Ultrastructural changes in biofilm forms of *staphylococci* cultivated in a mixed culture with lactobacilli. Regul Mechanisms Biosyst. 2017;8(1):98–103.

[CR26] Ma H, Li F, Niyitanga E, Chai X, Wang S, Liu Y. The odor release regularity of livestock and poultry manure and the screening of deodorizing strains. Microorganisms. 2021;9(12):2488. 34946090 10.3390/microorganisms9122488 PMC8705919

[CR27] Monchamp ME, Spaak P, Domaizon I, Dubois N, Bouffard D, Pomati F. Homogenization of lake cyanobacterial communities over a century of climate change and eutrophication. Nat Ecol Evol. 2018;2(2):317–24. 29230026 10.1038/s41559-017-0407-0

[CR28] Mu Q, Tavella VJ, Luo XM. Role of lactobacillus reuteri in human health and diseases. Front Microbiol. 2018;9:757. 29725324 10.3389/fmicb.2018.00757 PMC5917019

[CR29] Nie E, Gao D, Zheng G. Effects of lactic acid on modulating the ammonia emissions in co-composts of poultry litter with slaughter sludge. Biores Technol. 2020;315:123812. 10.1016/j.biortech.2020.123812 32682263

[CR30] Ning D, Deng Y, Tiedje J, Zhou J. A general framework for quantitatively assessing ecological stochasticity significance. Proc Natl Acad Sci. 2019;116(34):16892–8. 31391302 10.1073/pnas.1904623116 PMC6708315

[CR31] Qian X, Sun W, Gu J, Wang XJ, Zhang YJ, Duan ML, et al. Reducing antibiotic resistance genes, integrons, and pathogens in dairy manure by continuous thermophilic composting. Biores Technol. 2016;220:425–32. 10.1016/j.biortech.2016.08.101 27598571

[CR32] Reis JA, Paula AT, Casarotti SN, Penna ALB. Lactic acid bacteria antimicrobial compounds: characteristics and applications. Food Engineering Reviews. 2012;4(2):124–40.

[CR33] Ritchie ML, Romanuk TN. A meta-analysis of probiotic efficacy for gastrointestinal diseases. PloS One. 2012;7(4):e34938. 22529959 10.1371/journal.pone.0034938 PMC3329544

[CR34] Robertson S, Douglas P, Jarvis D, Marczyloa E. Bioaerosol exposure from composting facilities and health outcomes in workers and in the community: a systematic review update. Int J Hyg Environ Health. 2019;222(3):364–86. 30876873 10.1016/j.ijheh.2019.02.006

[CR35] Rybalchenko OV, Bondarenko VM, Orlova OG, Gusleva OR, Larionov IV, Fialkina SV. Disorganization of biofilms of clinical strains of *staphylococci* by metabolites of *lactobacilli*. Zh Mikrobiol Epidemiol Immunobiol. 2010;6:66–70. 21381381

[CR36] Sayara T, Basheer-Salimia R, Hawamde F, Sanchez A. Recycling of organic wastes through composting: process performance and compost application in agriculture. Agronomy-Basel. 2020;10(11):1838.

[CR37] Siedler S, Rau MH, Bidstrup S, Vento JM, Aunsbjerg SD, Bosma EF, et al. Competitive exclusion is a major bioprotective mechanism of lactobacilli against fungal spoilage in fermented milk products. Appl Environ Microbiol. 2020;86(7):e02312-19. 32005739 10.1128/AEM.02312-19 PMC7082583

[CR38] Starnawski P, Bataillon T, Ettema T, Jochum LM, Kjeldsen KU. Microbial community assembly and evolution in subseafloor sediment. Proc Natl Acad Sci. 2017;114(11):201614190. 10.1073/pnas.1614190114 PMC5358386 28242677

[CR39] Stegen JC, Lin X, Konopka AE, Fredrickson JK. Stochastic and deterministic assembly processes in subsurface microbial communities. ISME J. 2012;6:1653–64. 22456445 10.1038/ismej.2012.22 PMC3498916

[CR40] Tuchscherr L, Medina E, Hussain M, Voelker W, Heitmann V, Niemann S, et al. Staphylococcus aureus phenotype switching: an effective bacterial strategy to escape host immune response and establish a chronic infection. EMBO Mol Med. 2011;3(3):129–41. 21268281 10.1002/emmm.201000115 PMC3395110

[CR41] Wang C, Chang T, Yang H, Cui M. Antibacterial mechanism of lactic acid on physiological and morphological properties of *Salmonella Enteritidis*, *Escherichia coli* and *Listeria monocytogenes*. Food Control. 2015;47:231–6.

[CR42] Wery N. Bioaerosols from composting facilities-a review. Front Cell Infect Microbiol. 2014;4:42. 24772393 10.3389/fcimb.2014.00042 PMC3983499

[CR43] Wysocka I, Gbicki J, Namienik J. Technologies for deodorization of malodorous gases. Environ Sci Pollut Res. 2019;26(10):9409–34. 10.1007/s11356-019-04195-1 PMC6469639 30715695

[CR44] Xie WW, Li YP, Bai WY, Hou JL, Ma TF, Zeng XL, et al. The source and transport of bioaerosols in the air: a review. Front Environ Sci Eng. 2021;15(3):1–9. 10.1007/s11783-020-1336-8 PMC7876263 33589868

[CR45] Yan Z, Xu L, Li Z, Liu X, Wei X. Progress in research and application of controlling odor from livestock manure. Chin J App Environ Biol. 2014;20(2):322–7.

[CR46] Yong K. Btrim: a fast, lightweight adapter and quality trimming program for next-generation sequencing technologies. Genomics. 2011;98(2):152–3. 21651976 10.1016/j.ygeno.2011.05.009

[CR47] Zhang J, Chen M, Huang J, Guo X, Zhang Y, Liu D, et al. Diversity of the microbial community and cultivable protease-producing bacteria in the sediments of the Bohai Sea, Yellow Sea and South China Sea. PloS One. 2019;14(4):e0215328. 30973915 10.1371/journal.pone.0215328 PMC6459509

[CR48] Zhang X, Li SZ, Cheng WT, Zhao Y, Cui HY, Xie XY, et al. Oxytetracycline stress reconstruct the core microbial community related to nitrogen transformation during composting. Biores Technol. 2021a;319:124142. 10.1016/j.biortech.2020.124142 32987278

[CR49] Zhang Y, Dai Z, Zhou Z, Yin H, Meng D. Development of the yeast and lactic acid bacteria co-culture agent for atmospheric ammonia removing: Genomic features and on-site applications. Ecotoxicol Environ Saf. 2021b;218(6):112287. 33933812 10.1016/j.ecoenv.2021.112287

[CR50] Zhu F, Hong C, Wang W, Lyu H, Yao Y. A microbial agent effectively reduces ammonia volatilization and ensures good maggot yield from pig manure composted via housefly larvae cultivation. J Clean Prod. 2020;270(1):122373.

